# A Strap-Down Inertial Navigation/Spectrum Red-Shift/Star Sensor (SINS/SRS/SS) Autonomous Integrated System for Spacecraft Navigation

**DOI:** 10.3390/s18072039

**Published:** 2018-06-26

**Authors:** Zhaohui Gao, Dejun Mu, Yongmin Zhong, Chengfan Gu

**Affiliations:** 1 School of Automatics, Northwestern Polytechnical University, Xi’an 710072, China; mudejun@nwpu.edu.cn; 2 School of Engineering, RMIT University, Bundoora, Melbourne 3083, Australia; yongmin.zhong@rmit.edu.au; 3 Department of Mechanical Engineering, University of Melbourne, Parkville, Melbourne 3010, Australia; chengfan.gu@gmail.com

**Keywords:** spacecraft navigation, spectral red-shift, SINS/SRS/SS integrated navigation system, robust adaptive unscented particle filter

## Abstract

This paper presents a new Strap-down Inertial Navigation System/Spectrum Red-Shift/Star Sensor (SINS/SRS/SS) system integration methodology to improve the autonomy and reliability of spacecraft navigation using the spectrum red-shift information from natural celestial bodies such as the Sun, Jupiter and the Earth. The system models for SINS/SRS/SS integration are established. The information fusion of SINS/SRS/SS integration is designed as the structure of the federated Kalman filter to fuse the local estimations of SINS/SRS and SINS/SS integrated subsystems to generate the global state estimation for spacecraft navigation. A new robust adaptive unscented particle filter is also developed to obtain the local state estimations of SINS/SRS and SINS/SS integrated subsystems in a parallel manner. The simulation results demonstrate that the proposed methodology for SINS/SRS/SS integration can effectively calculate navigation solutions, leading to strong autonomy and high reliability for spacecraft navigation.

## 1. Introduction

Considerable research efforts have been dedicated to spacecraft navigation, resulting in various navigation techniques such as ground radio navigation, satellite navigation system, inertial navigation system (INS) and celestial navigation system [1,2]. Radio navigation is a non-autonomous navigation technique [3,4]. It is sensitive to external disturbances, since its navigation accuracy depends on the coverage area of ground stations and the propagation conditions of radio waves. The satellite navigation is an extension of the space-based radio navigation [5,6]. It is simple in implementation and highly accurate in positioning. However, its performance is vulnerable to human-induced disturbance. Consequently, the satellite navigation cannot achieve full autonomy. The Strap-down Inertial Navigation System (SINS) has a simple structure and strong autonomy, and is commonly used in vehicle navigation. Nevertheless, the SINS error accumulates over time, leading to biased or even divergent navigation solutions [7,8,9]. The celestial navigation, which uses the star sensor (SS) to derive spacecraft position and attitude, is also an autonomous navigation system. It can provide high-precision orientation information and inhibit electromagnetic interference [6,10]. However, it cannot directly measure spacecraft velocity and has a low data update rate. It also suffers from the limitation of SS, and thus cannot be used alone for positioning and navigation.

The spectrum red-shift (SRS) is a relatively new technology for autonomous navigation. Different from SS, SRS calculates the relative velocity between the spacecraft and the high-precision celestial bodies such as the Sun, Jupiter and the Earth. The velocity of the spacecraft can be obtained from the spectral information (celestial ephemeris) of the solar system, without requiring any information on ground radio and relying on spacecraft orbital dynamics equations. This method has the merits of simple implementation, high precision, strong autonomy and excellent real-time performance [11,12,13], leading to a promising solution to improve the autonomy of spacecraft navigation. However, spectrum signals can be temporarily interrupted by the occlusion of celestial bodies, and thus the navigation solution of SRS may be deteriorated or even divergent due to insufficient measurement information [14,15,16,17].

Given the complementary nature of SINS, SRS and SS, it is absolutely necessary to develop an integrated navigation system by integrating these sensors together to overcome their respective shortages, leading to an improved performance for spacecraft navigation. However, there has been very limited research on SINS/SRS/SS integrated navigation systems. Just recently, Wei et al. studied a Strapdown Inertial Navigation System/Spectral Red-Shift/Geomagnetic Navigation System (SINS/SRS/GNS) integrated navigation system [18]. However, the attitude accuracy of GNS is limited, being much lower than that of SS. This limited attitude accuracy of GNS also causes the attitude accuracy of SINS/SRS/GNS integration to be limited. GNS also involves an expensive computational process to search and match local geomagnetic maps and calculate local coordinates.

The essential concept of the SINS/SRS/SS integrated navigation system is multi-sensor data fusion. The federated Kalman filter (FKF) is a popular multi-sensor data fusion strategy in integrated navigation systems [1,19,20]. It conducts local and global filtering based on the principle of information sharing and also discards the dependence of local estimations via upper bounds [1,19,20].

However, the performance of FKF is heavily dependent on that of local filtering. At present, the extended Kalman filter (EKF), unscented Kalman filter (UKF) and particle filter (PF) are the typical filtering algorithms used for nonlinear systems. EKF is a suboptimal algorithm for nonlinear state estimation, where the system model is linearized by a Taylor expansion [21]. As the linearization process causes a significant error, the EKF solution may be biased or divergent [22]. EKF also requires the calculation of Jacobian matrix, which is difficult to achieve when measurement is strong nonlinear and systems noise is non-Gaussian. UKF is also a nonlinear filtering algorithm by approximating the probability density of state distribution based on unscented transform [21]. It reduces the linearization error of EKF and does not involve the cumbersome calculation of Jacobian matrix. However, it causes an extra computational load and is not stable in case of high-dimensional non-Gaussian systems. PF provides optimal Bayesian approximations of posterior distributions by Monte-Carlo simulation. It is suitable for strong nonlinear and non-Gaussian systems. However, it requires appropriate importance sampling, which is difficult to determine and thus may lead to degraded or divergent solutions. The central difference particle filter (CDPF) adopts the central difference Kalman filter to improve the importance sampling, leading to second-order accuracy for the mean and variance [18]. The Unscented Particle Filter (UPF) improves CDPF by implementing sampling points using unscented transformation to approximate the posterior density function for nonlinear systems. It can achieve the mean and variance in third-order accuracy. However, like PF, UPF still suffers from particle degradation [23].

This paper presents a new methodology for a SINS/SRS/SS autonomous integrated navigation system to improve the autonomy, reliability and accuracy of spacecraft navigation. The system models for SINS/SRS/SS integration are established based on the position and velocity of SRS and the attitude of SS. The information fusion for SINS/SRS/SS integration is designed as the structure of FKF, where the local state estimations of SIN/SRS and SINS/SS integrated subsystems are obtained independently by a robust adaptive unscented particle filter (RAUPF) and are further fused to generate the globally optimal state estimation for spacecraft navigation. This RAUPF adopts the concept of robust adaptive filtering in UPF to prevent particles from degeneracy. It uses the equivalent weight function and adaptive factor to improve the importance sampling resulted from unscented transformation based on the information of system state and measurement models. Simulation trails were performed to examine the efficacy of the presented methodology for SINS/SRS/SS integrated navigation system.

This paper is different from Wei’s work on SINS/SRS/GNS integration [18]. It focuses on SINS/SRS/SS integration, which is advantageous to SINS/SRS/GNS integration. Accordingly, the system models for integrated navigation in this paper are different from those in Wei’s work. Further, the filtering algorithm in this paper is also different from that in Wei’s work. This paper develops a RAUPF, while Wei’s work a robust adaptive CDPF (RACDPF), for local fusion. The RACDPF in Wei’s work reduces the computational load, which is caused by GNS for matching local geomagnetic maps and calculating local coordinates. However, since UPF has higher accuracy than CDPF, the RAUPF developed in this paper also has higher accuracy than RACDPF.

## 2. SRS Navigation

**Figure 1:**
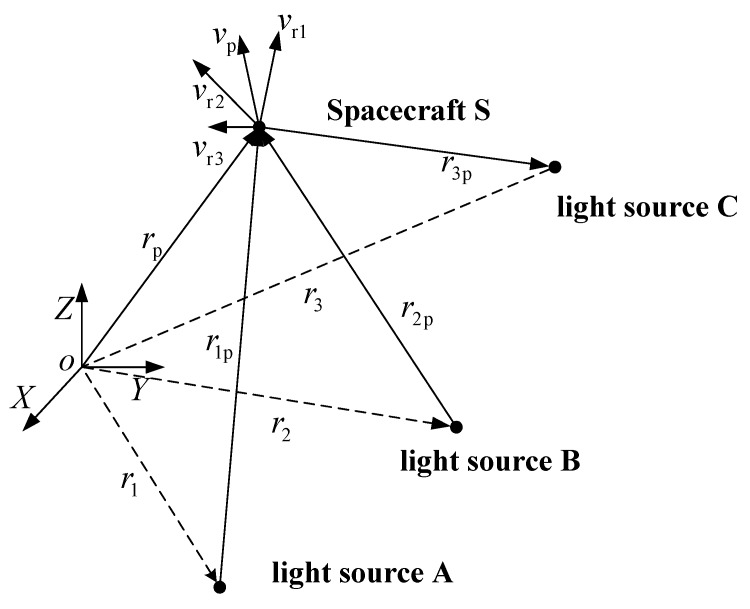
The principle of spectrum red-shift navigation.

The spacecraft spectrum red-shift autonomous navigation is based on the characteristics of the deep space environment. It treats the optical signals of celestial bodies in the solar system as navigation information sources. According to the Doppler effect, the frequency of the spectrum emitted from a celestial body is not equal to that received by the spacecraft, which varies with the spacecraft motion with reference to the celestial body. Assume there are three celestial bodies, i.e., three light sources, in the solar system. As shown in Figure 1, the velocity of the spacecraft in the inertial coordinate system can be obtained according to the measurement of the spectrum red-shift frequency based on the vector space theory. Accordingly, the position of the spacecraft in inertial coordinate system can be obtained by integrating the velocity over time.

When the spacecraft is moving relative to a celestial body, the relationship between the light wave frequencies $f_{m}$ and $f_{0}$ from the celestial body to the spacecraft and ground station can be expressed as:

(1) $$f_{m}=f_{0}\frac{\sqrt{1-{\left|v\right|}^{2}/c^{2}}}{1+\left|v\right|\cos\theta /c}$$

where $f_{m}$ and $f_{0}$ are the light wave frequencies from the celestial body to the spacecraft and ground station, respectively, v is the two-dimensional velocity vector of the spacecraft relative to the celestial body, θ is the angle between velocity vector v and the light wave vector from the celestial body to the spacecraft; |v|cosθ is the radial velocity, and c is the velocity of the celestial body in a vacuum.

According to Equation (1), the following relationship may be written:

(2) $$1+\left|v\right|\cos\theta /c=\frac{f_{0}}{f_{m}}\sqrt{1-{\left|v\right|}^{2}/c^{2}}$$

Equation (2) may be further written as:

(3) $$\left|v\right|\cos\theta =\frac{f_{0}}{f_{m}}\sqrt{c^{2}-{\left|v\right|}^{2}}-c$$

Equation (3) is actually the expression of the radial velocity:

(4) $$\begin{array}{l} v_{r}=\left|v\right|\cos\theta \\ \;=\frac{f_{0}}{f_{m}}\sqrt{c^{2}-{\left|v\right|}^{2}}-c \end{array}$$

Thus, for the case of three celestial bodies as shown in Figure 1, the light wave frequencies $f_{m1}$, $f_{m2}$ and $f_{m3}$ received by the spacecraft can be obtained from the light wave frequencies $f_{01}$, $f_{02}$ and $f_{03}$ received and measured by the ground station.

According to Equation (4), the radial velocity of the spacecraft relative to the three celestial bodies can be expressed as:

(5) $$\left\{ \begin{array}{l} v_{r1}=\frac{f_{01}}{f_{m1}}\sqrt{c^{2}-{\left|v_{p}\right|}^{2}}-c\\ v_{r2}=\frac{f_{02}}{f_{m2}}\sqrt{c^{2}-{\left|v_{p}\right|}^{2}}-c\\ v_{r3}=\frac{f_{03}}{f_{m3}}\sqrt{c^{2}-{\left|v_{p}\right|}^{2}}-c \end{array}\right.$$

where $v_{p}$ is the velocity vector of the spacecraft in the inertial coordinate system. By spatial geometry reasoning in relation to the three celestial bodies, the relationship between $v_{p}$ and $v_{r1}$, $v_{r2}$, $v_{r3}$ can be expressed as:

(6) $$\left\{ \begin{array}{l} v_{r1}=\left(v_{P}-v_{1}\right)\cdot u_{1}\\ v_{r2}=\left(v_{P}-v_{2}\right)\cdot \;u_{2}\\ v_{r3}=\left(v_{P}-v_{3}\right)\cdot \;u_{3} \end{array}\right.$$

where $v_{1}$, $v_{2}$ and $v_{3}$ are three celestial bodies’ velocities in the inertial frame. $u_{1}$, $u_{2}$ and $u_{3}$ are the unit vectors in the inertial frame, pointing to the spacecraft from each of the three celestial bodies.

The state equations in terms of the velocity vector and position vector can be described as:

(7) $$\left\{ \begin{array}{l} v_{P}=f\left(v_{1},v_{2},v_{3},u_{1},u_{2},u_{3},v_{r1},v_{r2},v_{r3}\right)\\ p_{P}=\int v_{P}dt \end{array}\right.$$

where f(⋅) describes the nonlinear function. Given the initial value, the velocity vector $v_{p}$ of the spacecraft in the inertial frame may be acquired via Equation (7). Then, the position vector $p_{P}$ can be obtained by integration.

## 3. System Models for SINS/SRS/SS Autonomous Integrated Navigation

The SINS/SRS/SS autonomous integrated navigation system consists of integrated SINS/SRS and SINS/SS subsystems. Its navigation coordinate system is chosen as East-North-Up (E-N-U) geographic coordinate system.

### 3.1. System State Equation

The system state vector is defined as:

(8) $$X\left(t\right)={\left[\begin{array}{ccccccccccccccc} \delta v_{E} & \delta v_{N} & \delta v_{U} & \delta \varphi & \delta \lambda & \delta h & \psi_{E} & \psi_{N} & \psi_{U} & \theta_{x} & \theta_{y} & \theta_{z} & \zeta_{x} & \zeta_{y} & \zeta_{z} \end{array}\right]}^{T}$$

where $\left(\delta v_{E},\delta v_{N},\delta v_{U}\right)$, (δφ,δλ,δh) and $\left(\psi_{E},\psi_{N},\psi_{U}\right)$ are the errors in velocity, position and attitude; $\left(\theta_{x},\theta_{y},\theta_{z}\right)$ is the constant bias of the gyros; and $\left(\zeta_{x},\zeta_{y},\zeta_{z}\right)$ the zero bias of the accelerometers.

The system state equation of SINS/SRS/SS integration is described by:

(9) $$\dot{X}\left(t\right)=f\left(X\left(t\right)\right)+G(t)W(t)$$

where f(⋅) is a nonlinear function given by Equation (10) [19], X(t) is the system state vector, and W(t) is the system noise.

(10) $$\begin{array}{l} f\left(X\left(t\right)\right)=\\ \;[\begin{array}{l} C_{\omega }^{-1}\left[\left(I-C_{n}^{c}\right){\hat{\omega }}_{in}^{n}+C_{n}^{c}\delta \omega_{in}^{n}-C_{b}^{c}\delta \omega_{ib}^{b}\right]\\ \left[I-{\left(C_{n}^{c}\right)}^{T}\right]C_{b}^{c}{\hat{f}}_{sf}^{b}+{\left(C_{n}^{c}\right)}^{T}C_{b}^{c}\delta f_{sf}^{b}\\ -\left(2\delta \omega_{ie}^{n}+\delta \omega_{en}^{n}\right)\times V-\left(2{\hat{\omega }}_{ie}^{n}+{\hat{\omega }}_{en}^{n}\right)\times \delta V\\ +\left(2\omega_{ie}^{n}+\omega_{en}^{n}\right)\times \delta V+\delta g\\ \frac{v_{N}}{R_{M}+h}-\frac{\left(v_{N}-\delta v_{N}\right)}{\left(R_{M}-\delta R_{M}\right)+\left(h-\delta h\right)}\\ \frac{v_{E}\sec\varphi }{R_{N}+h}-\frac{\left(v_{E}-\delta v_{E}\right)\sec\left(\varphi -\delta \varphi \right)}{\left(R_{N}-\delta R_{N}\right)+\left(h-\delta h\right)}\\ \delta v_{U}\\ 0_{1\times 7} \end{array}] \end{array}$$

where $C_{\omega }^{}$ is the Euler platform error angel matrix; $C_{n}^{c}$ is the transformation matrix from the navigation (n) to computer (c) frames in terms of attitude; $C_{b}^{c}$ is the transformation matrix from the body (b) to computer (c) frames in terms of attitude; ${\hat{\omega }}_{ie}^{n}$ and $\omega_{ie}^{n}$ are the projections of the actual and ideal values of angular velocity into (n) from the Earth coordinate system (e) to the inertial coordinate system (i); $\delta \omega_{in}^{n}$ and $\delta \omega_{ib}^{b}$ are the calculation errors of $\omega_{in}^{n}$ and $\omega_{ib}^{b}$; ${\hat{f}}_{sf}^{b}$ and $\delta f_{sf}^{b}$ are the real accelerometer’s force and its associated error; V and δV are the real velocity and its associated error; δg is the error of gravity acceleration; φ and h are the latitude and altitude values; and $R_{M}$ and $R_{N}$ are the radii of curvatures of local meridian and prime vertical.

The noise coefficient matrix is defined as:

(11) $$G\left(t\right)={\left[\begin{array}{cc} \begin{array}{cc} C_{b(3\times 3)}^{c} & 0_{\left(3\times 3\right)}\\ 0_{\left(3\times 3\right)} & C_{b(3\times 3)}^{c} \end{array} & 0_{\left(9\times 7\right)}\\ 0_{\left(10\times 6\right)} & I_{\left(7\times 7\right)} \end{array}\right]}_{\left(16\times 13\right)}$$

### 3.2. Measurement Equation of SINS/SRS Integrated Subsystem

**Figure 2:**
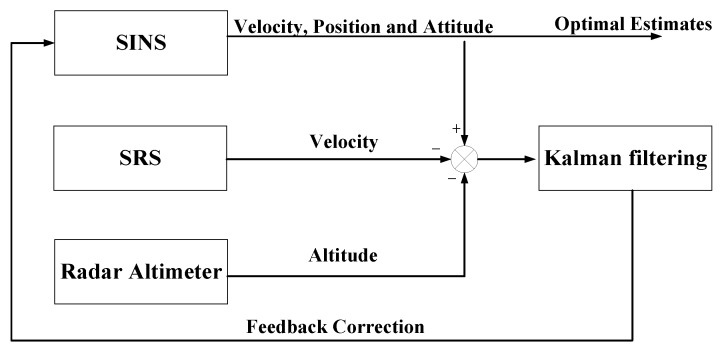
The integrated principle of SINS/SRS integrated subsystem.

The architecture of SINS/SRS integrated subsystem is shown in Figure 2. In order to overcome the drawback that the velocity error of SINS is accumulated in time series, the velocity information from SRS is used to correct the velocity error of SINS. Further, a radar altimeter is used to provide the altitude information to correct the altitude channel of SINS.

The measurement of SINS/SRS integrated subsystem can be chosen as the difference between the velocities of SINS and SRS as well as the difference of altitude between the radar altimeter and SINS.

Suppose the spacecraft velocities obtained by SRS and SINS are $V_{SRS}=\left(v_{SE},v_{SN},v_{SU}\right)$ and $V_{SINS}=\left(v_{E},v_{N},v_{U}\right)$. The difference of spacecraft velocity between SRS and SINS is defined as:

(12) $$\Delta Z_{v}=\left[\begin{array}{c} \Delta Z_{S1}\\ \Delta Z_{S2}\\ \Delta Z_{S3} \end{array}\right]=\left[\begin{array}{c} v_{E}-v_{SE}\\ v_{N}-v_{SN}\\ v_{U}-v_{SU} \end{array}\right]=H_{v}X\left(t\right)+V_{v}\left(t\right)$$

where $V_{v}$ is the velocity measurement noise matrix, and $H_{v}={\left[\begin{array}{cc} I_{3\times 3} & 0_{3\times 12} \end{array}\right]}^{T}$ is the measurement matrix.

Further, the difference of spacecraft altitude between the radar altimeter and SINS can be described as:

(13) $$\Delta Z_{h}=\left[h_{SINS}-h_{H}\right]=H_{h}X\left(t\right)+V_{h}\left(t\right)$$

where $h_{SINS}$ and $h_{H}$ are the spacecraft altitudes obtained by SINS and the radar altimeter; $V_{h}\left(t\right)$ is the measurement noise matrix of spacecraft altitude; and $H_{h}={\left[\begin{array}{ccc} 0_{1\times 5} & 1 & 0_{1\times 9} \end{array}\right]}^{T}$ is the measurement matrix.

Combining Equation (12) with Equation (13), the measurement equation of SINS/SRS integrated subsystem is established as:

(14) $$\Delta Z_{1}\left(t\right)=\left[\begin{array}{c} H_{v}\\ H_{h} \end{array}\right]X\left(t\right)+\left[\begin{array}{c} V_{v}\left(t\right)\\ V_{h}\left(t\right) \end{array}\right]=H_{1}\left(t\right)X\left(t\right)+V_{1}\left(t\right)$$

### 3.3. Measurement Equation of SINS/SS Integrated Subsystem

**Figure 3:**
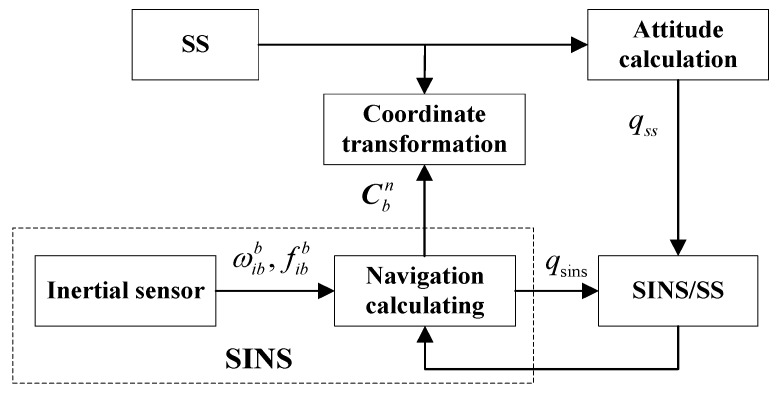
The framework of SINS/SS integrated subsystem.

Figure 3 shows the framework of SINS/SS integrated subsystem, where the spacecraft attitude obtained from SS is applied to correct the attitude error of SINS. The difference of spacecraft attitude between SS and SINS is taken as the measurement of SINS/SS integrated subsystem. The measurement equation is given by:

(15) $$\Delta Z_{2}\left(t\right)=H_{2}X\left(t\right)+V_{2}\left(t\right)$$

where $H_{2}=\left[\begin{array}{ccc} 0_{{}_{3\times 6}} & I_{3\times 3} & 0_{{}_{3\times 6}} \end{array}\right]$, and $V_{2}(t)$ is the measurement noise corresponding to the measurement error of SS.

## 4. Information Fusion for SINS/SRS/SS Integrated Navigation

In this section, a fusion framework is designed as the structure of FKF for INS/SRS/SS integration, where the local state estimations of SINS/SRS and SINS/SS integrated subsystems are obtained by a local filter in a parallel manner and further fused to generate the global state estimation for spacecraft navigation. The local filter is implemented by the proposed RAUPF to obtain the local state estimations of SINS/SRS and SINS/SS integrated subsystems.

### 4.1. Fusion Framework

**Figure 4:**
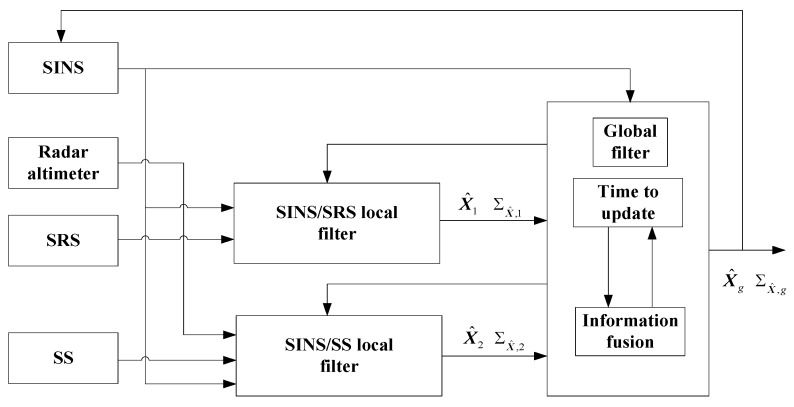
The fusion principle of SINS/SRS/SS integrated navigation system.

As a multi-sensor system, information fusion is a key element in SINS/SRS/SS integration to achieve optimal state estimation. In this paper, the fusion framework of SINS/SRS/SS integration is designed as the structure of FKF. As shown in Figure 4, the two local filters are implemented by the proposed RAUPF to calculate the locally optimal estimations ${\hat{X}}_{i}$ and the corresponding error covariance matrices $\Sigma_{\hat{X},i}(i=1,2)$ of SINS/SRS and SINS/SS integrated subsystems in a parallel manner. Subsequently, the two local optimal estimations are fused by using the federated filtering technology [6,24,25,26] to obtain the globally optimal state estimation ${\hat{X}}_{g}$ and the associated error covariance matrix $\Sigma_{\hat{X},g}$, i.e.,

(16) $$\{\begin{array}{c} \Sigma_{\hat{X},g}={\left(\Sigma_{\hat{X},1}^{-1}+\Sigma_{\hat{X},2}^{-1}\right)}^{-1}\\ {\hat{X}}_{g}=\Sigma_{\hat{X},g}(\Sigma_{\hat{X},1}^{-1}{\hat{X}}_{1}+\Sigma_{\hat{X},2}^{-1}{\hat{X}}_{2}) \end{array}$$

where the local state estimations of SINS/SRS and SINS/SS integrated subsystems are generated by the two local filters in a parallel manner and are subsequently fused to generate the global state estimation for INS/SRS/SS integrated navigation.

Finally, the SINS error is rectified by ${\hat{X}}_{g}$ in real time. As mentioned previously, since the altitude channel of SINS is divergent and neither SRS nor SS can output the spacecraft altitude, the radar altimeter is used to correct the SINS altitude channel to suppress the divergence in the altitude of SINS.

### 4.2. Robust Adaptive Unscented Particle Filter

Consider the following nonlinear system [27,28]:

(17) $$\left\{ \begin{array}{l} x_{k}=f(x_{k-1},w_{k-1})\\ z_{k}=h(X_{k},V_{k}) \end{array}\right.$$

where $x_{k}\in R^{n}$ is the state vector of the system at time *k*, $z_{k}\in R^{n}$ is the measurement vector, $w_{k}\in R^{n}$ is the system noise with variance $R_{k}$, $V_{k}\in R^{n}$ is the measurement noise with variance $Q_{k}$, both f(⋅) and h(⋅) are a nonlinear function, and the sampling time is k=0,1,⋯,N. The procedure of the proposed RAUPF includes the following steps:

(i) Initialization. Draw N particles according to the initial state and corresponding error covariance. For time k=0, let $x_{{}_{0}}^{i}\sim p(x_{o})$, i=1,2,⋯,N, and set the initial weight as $w_{{}_{0}}^{i}=1/N$.
(ii) For time k=1,2,⋯,N, conduct the following steps:
  (a) Calculate the equivalent weight matrix $\bar{P}$ and the adaptive factor α. In order to enhance the robustness of measurement, the IGG algorithm [29,30] is adopted to construct equivalent weight matrix $\bar{P}$ as the following decreasing function:
    (18) $$\bar{P}=diag({\bar{P}}_{1},{\bar{P}}_{2},\cdots ,{\bar{P}}_{k})\;{\bar{P}}_{k}=(\begin{array}{ccc} p_{k} & & \left|\varepsilon_{k}\right|\leq k_{0}\\ p_{k}\frac{k_{0}}{\left|\varepsilon_{k}\right|} & & k_{0}<\left|\varepsilon_{k}\right|\leq k_{1}\\ 0 & & \left|\varepsilon_{k}\right|>k_{1} \end{array}$$
    In some cases, the equivalent weight matrix can be also defined as [31]:
    (19) $${\bar{P}}_{k}=(\begin{array}{ccc} p_{k} & & \left|\varepsilon_{k}\right|\leq k_{0}\\ p_{k}\frac{k_{0}}{\left|\varepsilon_{k}\right|}\frac{{\left(k_{1}-\left|\varepsilon_{k}\right|\right)}^{2}}{{\left(k_{1}-k_{0}\right)}^{2}} & & k_{0}\leq \left|\varepsilon_{k}\right|<k_{1}\\ 0 & & k_{1}\leq {\left|\varepsilon_{k}\right|}_{} \end{array}$$
    where $k_{0}\in (1,1.5)$, $k_{1}\in (3,8)$, $\varepsilon_{k}$ is the residual vector of measurement $z_{k}$, and ${\hat{x}}_{k}$ is the current state estimate.
    The adaptive factor is selected as:
    (20) $$\alpha =(\begin{array}{ccc} 1 & & \left|\Delta {\tilde{x}}_{k}\right|\leq c_{0}\\ \frac{c_{0}}{\left|\Delta {\tilde{x}}_{k}\right|}\frac{{\left(c_{1}-\left|\Delta {\tilde{x}}_{k}\right|\right)}^{2}}{{\left(c_{1}-c_{0}\right)}^{2}} & & c_{0}\leq \left|\Delta {\tilde{x}}_{k}\right|<c_{1}\\ 0 & & c_{1}\leq {\left|\Delta {\tilde{x}}_{k}\right|}_{} \end{array}$$
    where $c_{0}\in (1,1.5)$, $c_{1}\in (3,8)$, $\Delta {\tilde{x}}_{k}=\frac{\left\|{\hat{x}}_{k}-{\bar{x}}_{k}\right\|}{\sqrt{tr(\sum {\bar{x}}_{k})}}$, tr(⋅) is the trace of the matrix, ${\bar{x}}_{k}$ is the state prediction, and ${\hat{x}}_{k}={\left(A_{k}^{T}{\bar{P}}_{k}A_{k}+\alpha P_{{\bar{x}}_{k}}\right)}^{-1}(A_{k}^{T}{\bar{P}}_{k}l_{k}+\alpha P_{{\bar{x}}_{k}}{\bar{x}}_{k})$.
  (b) Calculate the Sigma points. Update the particles $\left\{x_{k-1}^{i},p_{k-1}^{i}\right\}$ using UKF to obtain $\left\{{\bar{x}}_{k}^{i},p_{k}^{i}\right\}$, where ${\bar{x}}_{k}^{i}$ satisfies $q(x_{k}^{i}|x_{k-1}^{i},z_{k})=N({\bar{x}}_{k}^{i},p_{k}^{i})$. Taking ${\bar{x}}_{k}^{i}$ as a new sample, 2N+1 Sigma points are selected as [27,28]:
    (21) $$\chi_{k-1}^{i}=[{\bar{x}}_{k-1}^{i},{\bar{x}}_{k-1}^{i}+\sqrt{(N+\lambda )P_{k-1}^{i}},{\bar{x}}_{k-1}^{i}-\sqrt{(N+\lambda )P_{k-1}^{i}}]$$
    where $\lambda =\varepsilon^{2}(n+\rho )$ represents the second order scale factor, *n* is the dimension of the system state, ρ is the adjustment coefficient, N denotes the quantity of particle samples, and ε is the factor to measure the sample distribution in regard to the mean of the predicted state. Subsequently, UKF is used to update the particles.
(iii) Calculate the weights $w_{k}^{i}=w_{k-1}^{i}\frac{p(y_{k}|{\bar{x}}_{k}^{i^{*}})p({\bar{x}}_{k}^{i^{*}}|{\bar{x}}_{k-1}^{i^{*}})}{q({\bar{x}}_{k}^{i^{*}}|{\bar{x}}_{k-1}^{i^{*}},z_{k})}$, and normalize them as ${\tilde{w}}_{k}^{i}=w_{k}^{i}/\sum_{i=1}^{n}w_{k}^{i}$. In the above formula, ${\bar{x}}_{k}^{i}{}^{*}={\bar{x}}_{k|k-1}^{i},+K_{k}^{*}(z_{k}^{}-{\bar{z}}_{k|k-1}^{i})$, $K_{k}^{*}=P_{x_{k}l_{k}}{\bar{P}}_{l_{k}l_{k}}^{-1}$, and ${\bar{z}}_{k|k-1}^{i}=\sum_{j=0}^{2N}W_{j}^{m}z_{j,k|k-1}^{i}$.
(iv) Calculate the normalized estimate ${\hat{N}}_{eff}=1/\sum_{i=1}^{N}{\left({\tilde{w}}_{{}_{k}}^{i}\right)}^{2}$. The particle degradation can be measured with the value of ${\hat{N}}_{eff}$. If ${\hat{N}}_{eff}$ is smaller, the particle degradation will be worse. In case particles are severely degraded, once can resample the posterior density and further allocate the same factor $\frac{1}{M}$ to every newly sampled particle.
(v) Calculate the state estimation ${\hat{x}}_{k}^{*}=\sum_{i=1}^{N}{\tilde{w}}_{k}^{i}{\bar{x}}^{i}{{}_{k}}^{*}$. Then, repeat the above steps (ii)–(iv) for the next time point.

In the above steps, in order to achieve a more effective distribution function for the process of importance sampling, the particles resulted from unscented transformation are governed by equivalent weight matrix $\bar{P}$, N and α.

## 5. Simulations and Analysis

The presented methodology was assessed by conducting simulations for SINS/SRS/SS integration in terms of the flight of a spacecraft. Comparison analysis with SINS as well as SINS/SRS and SINS/SS integrated subsystems were also conducted to demonstrate the efficiency of the proposed methodology for SINS/SRS/SS integration. Further, simulations and comparison analysis with EKF, UKF, PF and UPF were also conducted to evaluate the RAUPF effectiveness for SINS/SRS/SS integration.

**Figure 5:**
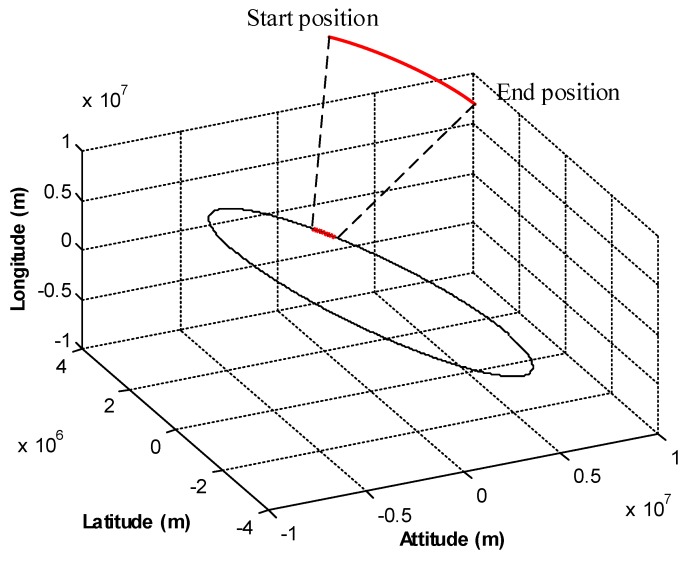
Flight trajectory of the spacecraft.

The navigation coordinate system is the East-North-Up geocentric coordinate system. Assume the spacecraft orbits the Earth, and the orbit parameters are described in Table 1. A flight period of 1000 s was selected for the simulation test, where the initial position was (2,207,542 m, 3,393,318 m, −2,194,259 m), and the end position was (1,229,267 m, 3,515,079 m, −1,194,458 m). The flight trajectory is shown in Figure 5.

In the process of simulation, the SINS measurements, namely, the angular velocity increments of the gyros and the specific force of the accelerometers, are generated according to the flight trajectory of the spacecraft and the parameters of Earth rotation. The SRS measurements are generated by ASTM G173-03 Reference Spectra derived from SM-ARTS [32]. The SS measurements are obtained from the astronomical ephemeris information and the flight trajectory of the spacecraft.

The initial alignment error of SINS is zero. The initial position error, initial velocity error and initial attitude error of the spacecraft are (10 m, 10 m, 10 m), (1 m/s, 1 m/s, 1 m/s) and ($1^{\prime }$, $1^{\prime }$, $1^{\prime }$). The unscented transformation parameters are α=0.5 and β=2. The adaptive factor calculation parameters are $c_{0}=1$ and $c_{1}=3.5$. The equivalent weight matrix calculation parameters are $k_{0}=1$ and $k_{1}=4.2$. The number of particles is M=200. The sensor parameters used in the simulation test are shown in Table 2.

### 5.1. Performances of SINS/SRS and SINS/SS Integrated Subsystems

**Figure 6:**
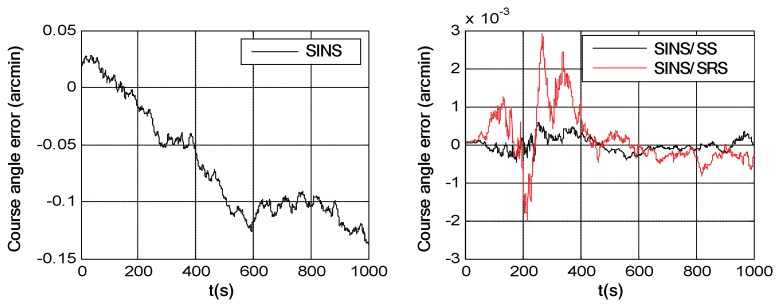
Course angle errors of SINS as well as SINS/SRS and SINS/SS integrated subsystems.

**Figure 7:**
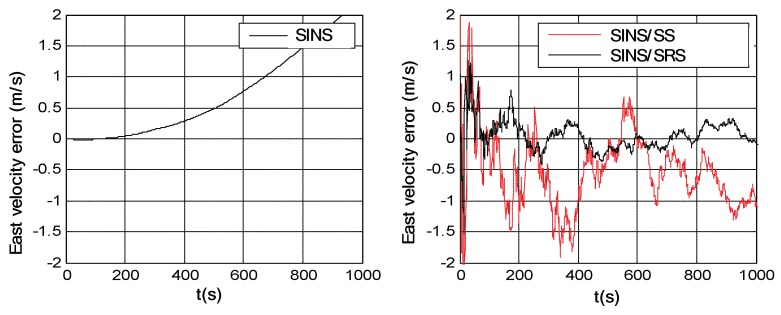
East velocity errors of SINS as well as SINS/SS and SINS/SRS integrated subsystems.

**Figure 8:**
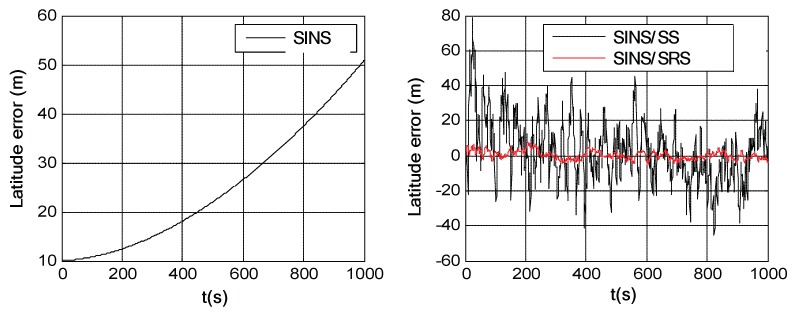
Latitude errors of SINS as well as SINS/SS and SINS/SRS integrated subsystems.

Simulation trials were conducted to evaluate the navigation performances of the SINS/SRS and SINS/SS integrated subsystems. The solutions of the SINS/SRS and SINS/SS integrated subsystems were achieved under the same conditions using RAUPF, and were further compared with the flight trajectory as reference to calculate their individual navigation errors. For comparison analysis, the SINS navigation error was also calculated in the simulation trials. Figure 6, Figure 7 and Figure 8 show the course angle errors, East velocity errors and latitude errors of SINS, and SINS/SRS and SINS/SS integrated subsystems. Table 3 summaries the root mean square errors (RMSEs) of SINS/SRS and SINS/SS integrated subsystems.

From the above simulation results, it is obvious that the standalone SINS cannot provide a high-precision navigation solution. Its attitude error, velocity error and position error are accumulated and divergent with time. Further, for SINS/SS integrated subsystem, SS can effectively correct the attitude error of SINS with its highly accurate attitude. However, it cannot effectively correct the position error of SINS. SINS/SRS integrated subsystem is the opposite of SINS/SS integrated subsystem, where SRS can effectively correct the velocity error of SINS with its high-accurate velocity, but it cannot effectively correct the attitude error of SINS. Thus, neither SINS/SS nor SINS/SRS integrated system can provide reliable solutions for spacecraft navigation. 

### 5.2. Performance of SINS/SRS/SS integrated navigation system

**Figure 9:**
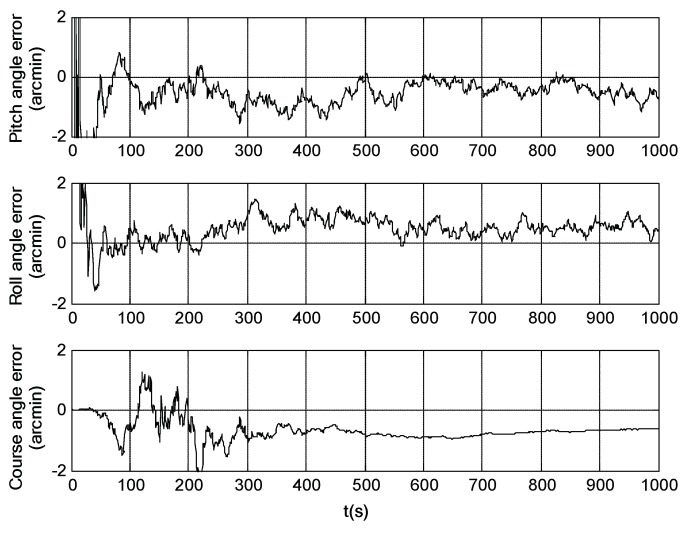
Attitude error of SINS/SRS/SS integrated navigation system.

**Figure 10:**
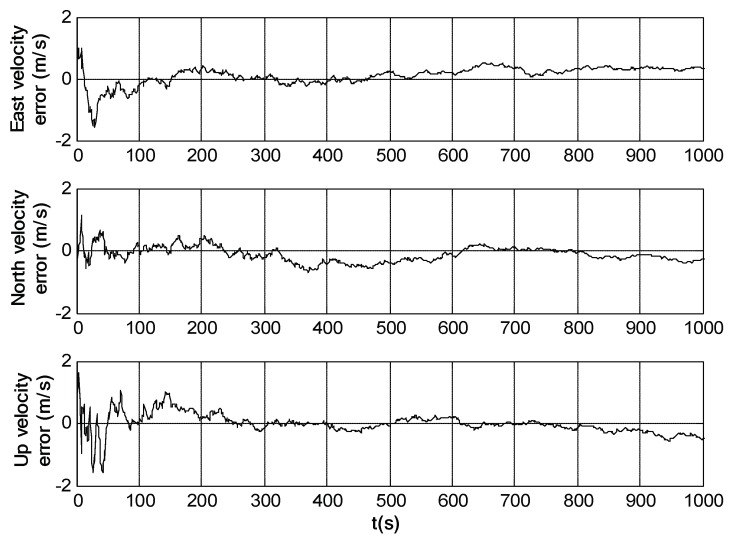
Velocity error of SINS/SRS/SS integrated navigation system.

**Figure 11:**
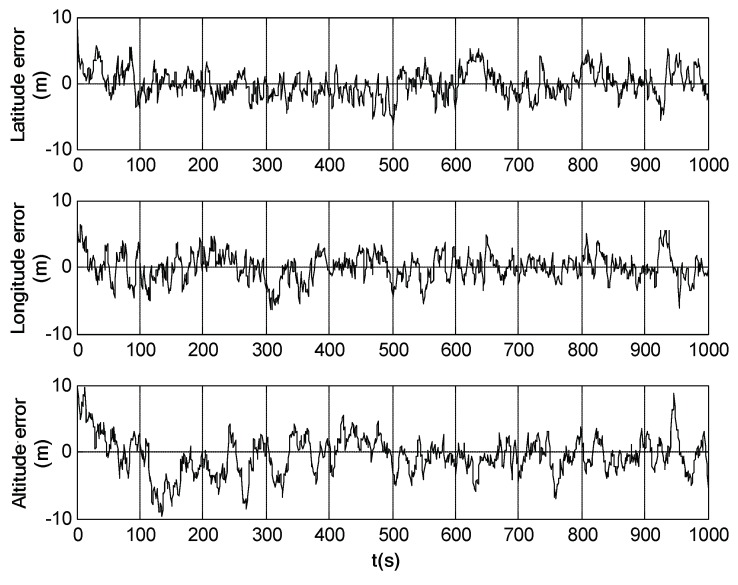
Position error of SINS/SRS/SS integrated navigation system.

Simulation trials were further conducted under the same conditions as those in Section 5.1 to evaluate the performance of SINS/SRS/SS integration. The simulation results are shown in Figure 9, Figure 10 and Figure 11. Table 4 shows the SINS/SRS/SS integration errors.

It can be seen from Figure 9, Figure 10 and Figure 11 that the attitude, velocity and position errors of SINS/SRS/SS integration is within 1′, 0.6m/s and 5m. This demonstrates that SINS/SRS/SS integration overcomes the disadvantages of single navigation systems by combining the advantages of SINS, SRS and SS, leading to the improved navigation accuracy and reliability.

### 5.3. Performance of RAUPF

Simulation trials were also conducted under the same conditions to evaluate the performance of the proposed RAUPF in comparison with EKF, UKF, PF and UPF for SINS/SRS/SS integration. For PF, UPF and RAUPF, three different particle numbers (M=100, M=200 and M=400) were used in the simulation trials.

**Figure 12:**
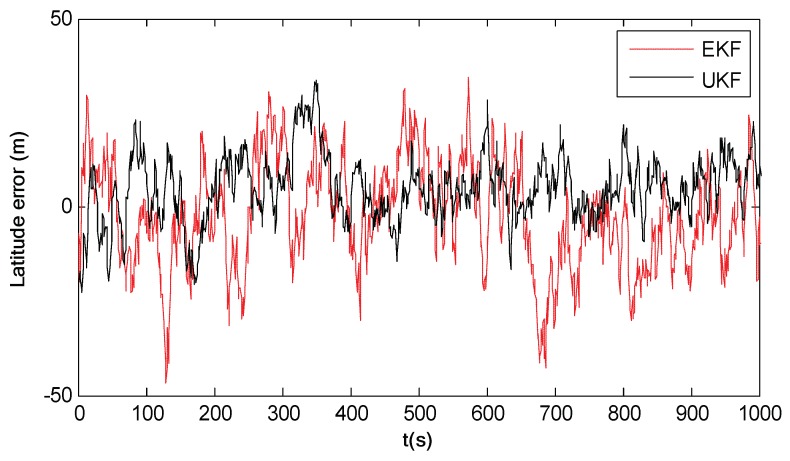
Latitude errors of EKF and UKF.

Figure 12 shows the latitude errors of EKF and UKF. It can be seen that the estimation accuracy of EKF is lower than that of UKF. This is because the linearization of the nonlinear model in EKF causes a large error to the state estimation. Although UKF improves the filtering accuracy of EKF, the improved accuracy is still limited. The reason is that UKF approximates the posterior probability distribution of the system state using the Gaussian distribution. When the posterior probability distribution of the system state is non-Gaussian, which is the case of the simulation test, the UKF performance will be significantly degraded. Therefore, both EKF and UKF have limited navigation accuracy for the spacecraft navigation.

**Figure 13:**
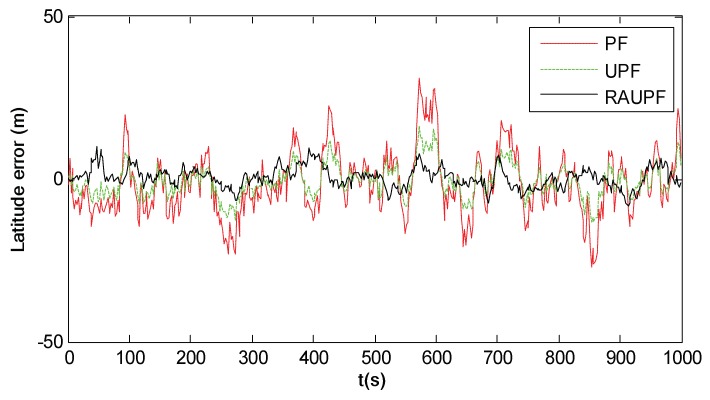
Latitude errors of PF, UPF and RAUPF (M=200).

Figure 13 shows the latitude errors of PF, UPF and RAUPF, where the particle number is M=200. Comparing Figure 13 with Figure 12, it is evident that all three particle filters (PF, UPF and RAUPF) have higher accuracy than both EKF and UKF. This is mainly because these three particle filters use samples to describe the a priori and a posteriori information, thus discarding the constraint that random samples must satisfy a Gaussian distribution. Further, it can also be seen that RAUPF has much higher accuracy than PF and UPF. This is because RAUPF uses the equivalent weight function and adaptive factor to control particle samples based on system state and measurement models to improve the importance sampling, leading to the enhanced accuracy.

Table 5 summaries the RMSEs of the above filters, where the RMSEs of PF, UPF and RAUPF are subject to three different particle numbers, i.e., M=100, M=200 and M=400. It can be seen that even with the small number of particles (M=100), all three particle filters (PF, UPF and RAUPF) still have higher accuracy than both EKF and UKF.

## 6. Conclusions

This paper presents a new methodology of SINS/SRS/SS integration for spacecraft navigation. It establishes the system models based on the position and velocity of SRS and the attitude of SS for SINS/SRS/SS integration. It also develops an information fusion framework for SINS/SRS/SS integration, where the local state estimations of SINS/SRS and SINS/SS subsystems are obtained by RAUPF and are further fused to generate the globally optimal state estimation for spacecraft navigation. This RAUPF uses the equivalent weight function and adaptive factor to improve the importance sampling, thus leading to the enhanced accuracy of state estimation. The simulation results demonstrate that the proposed methodology for SINS/SRS/SS integration realizes the globally optimal fusion of SINS/SRS and SINS/SS subsystems based on the locally optimal fusion results of each subsystem, thus effectively improving the autonomy, reliability and accuracy for spacecraft navigation.

Future work will focus on improvement of the proposed methodology. By adopting advanced artificial intelligence such as deep learning neural networks, genetic algorithms and pattern recognitions, the proposed methodology will be improved to intelligently characterize errors and uncertainties and further automatically inhibit their disturbances on state estimation in the fusion process.

## Figures and Tables

**Table 1:** The parameters of the spacecraft.

Orbit Parameters	Value
Semi-major axis	7128.042335 km
Eccentricity	0.001169
Orbit inclination	29.268°
Right ascension of ascending node	341.24°
Argument of perigee	347.128°
True anomaly	232.57°
Orbit cycle	49755 s

**Table 2:** The sensor parameters in the simulation test.

Sensor Parameters	Values
Gyro constant bias	0.02°/h
Gyro random drift	$0.005{}^{\circ}/\sqrt{h}$
Accelerometer constant bias	0.05 mg
Accelerometer random drift	$0.005\;\mathrm{mg}/\sqrt{h}$
Radar Altimeter measurement accuracy	5 m
SS measurement accuracy	$20^{\prime \prime }$
Spectral redshift estimation accuracy	Standard deviation ≤ $1.8\times 10^{-9}$

**Table 3:** Errors of SINS/SRS and SINS/SS integrated subsystems.

Error Type		Integrated Subsystem	
		SINS/SS	SINS/SRS
Attitude RMSE (′)	Pitch angle	0.886	2.7325
	Roll angle	0.86768	2.1698
	Course angle	1.0858	5.102
Velocity RMSE (m/s)	East	0.4945	0.1832
	North	0.5147	0.1987
	Up	0.5124	0.1963
Position RMSE (m)	Longitude	23.1405	2.7173
	Latitude	33.7497	2.6735
	Altitude	2.3447	2.3895

**Table 4:** Positioning errors of SINS/SRS/SS integrated navigation system.

Parameter		RMSE
Attitude (′)	Pitch	0.8399
	Roll	0.8431
	Course	0.8470
Position (m)	Longitude	2.6401
	Latitude	2.4153
	Altitude	2.5762
Velocity (m/s)	East	0.1911
	North	0.1962
	Up	0.1951

**Table 5:** The simulation results of the filtering algorithms.

Filtering Algorithms	Position RMSEs/m		
	M=100	M=200	M=400
EKF	-	14.7298	-
UKF	-	9.0291	-
PF	7.3213	6.4402	5.7090
UPF	5.9115	5.0808	4.3818
RAUPF	3.1862	2.4610	2.0954
